# Clustered Basic Amino Acids of the Small Sendai Virus C Protein Y1 Are Critical to Its Ran GTPase-Mediated Nuclear Localization

**DOI:** 10.1371/journal.pone.0073740

**Published:** 2013-08-09

**Authors:** Takashi Irie, Asuka Yoshida, Takemasa Sakaguchi

**Affiliations:** Department of Virology, Institute of Biomedical & Health Sciences, Hiroshima University, Hiroshima, Japan; Whitehead Institute, United States of America

## Abstract

The Sendai virus (SeV) C proteins are shown to exert multiple functions during the course of infection. Perhaps reflecting their many functions, they occur at multiple sites of the cell. In this study, we focused on the nuclear-localizing ability of the smaller C protein, Y1, and found that this translocation is mediated by Ran GTPase but not by passive diffusion, and that basic residues within the 149-157 amino acid region are critical for that. The mechanism of inhibition of interferon (IFN)-signaling seemed to differ between the C and Y1 proteins, since deletion of 12 C-terminal amino acids resulted in a loss of the function for the C but not for the Y1 protein. The ability of Y1 mutants to inhibit IFN-α-induced, ISRE-driven expression of a reporter gene almost paralleled with that to localize in the nucleus. These results suggest that nuclear localization of the Y1 protein might be important for the inhibitory effect on type-I IFN-stimulated gene expression.

## Introduction

Sendai virus (SeV; mouse parainfluenza virus type I) is a prototype of the family *Paramyxoviridae* of the order *Mononegavirales* which includes a number of important and ubiquitous disease-causing viruses of humans and animals, such as measles virus, parainfluenza viruses, mumps virus, Nipah virus, human metapneumovirus, canine distemper virus, and rinderpest virus [1]. SeV contains a nonsegmented, negative-stranded RNA genome of 15,384 nucleotides encoding six structural proteins, N, P, M, HN, F, and L, tandemly, in this order [2]. The P gene of paramyxoviruses is unique in producing more than one polypeptide species, and at least seven polypeptides are expressed from the SeV P gene: in addition to the P protein, four C proteins (C’, C, Y1, and Y2) are translated from start codons in the +1 open reading frame (ORF) relative to that of the P protein, and proteins V and W are produced from the altered P ORF with the insertion of one or two G residues at a specific position of the mRNA, respectively, during transcription [2]. These proteins are known as “accessory” proteins, because they are inessential for minimal viral growth in cultured cells. However, they have been found to play important roles in viral growth and pathogenicity [1,2].

The SeV C proteins have been shown to have multiple functions during viral replication in cell cultures (*in vitro*) as well as in mice (*in vivo*), such as counteraction against host innate immunity, inhibition of virus-induced apoptosis, regulation of polarized viral RNA synthesis, and promotion of efficient viral assembly and budding [3–9]. However, their best-characterized function is to counteract the Jak/STAT signaling pathway after stimulation by type I interferons (IFNs), resulting in a lack of activation of IFN-stimulated genes (ISGs) and establishment of an antiviral state in the infected cell [3,5,10–13]. This function has been reported to be accomplished by inhibition of tyrosine phosphorylation of cellular signal transducer and activator of transcription (STAT) 2 through physical interaction with STAT1, and mapped to the C-terminal half (amino acids [a.a.] 99-204) of the C protein [12–16]. The longer C proteins (C’ and C) have been reported to induce STAT1 instability in certain kinds of cells such as mouse embryonic fibroblasts (MEFs) and human fibrosarcoma 2fTGH cells, thereby preventing the establishment of an antiviral state in an IFN-α/β signaling-independent manner [11]. The longer C proteins also induce the IFN-independent phosphorylation of STAT1, although the significance of this to viral replication and pathogenicity is unknown [15,17,18]. The latter two functions are mapped to within 23 N-terminal amino acids of the 204-residue C protein [19]. In addition, the C proteins have been shown to prevent not only IFN-α/β, but also IFN-γ signaling, and to suppress IFN-β production through unknown mechanisms [3,5,13,16,20].

During viral replication, viral proteins must be located at appropriate sites in the host cell to function properly. In this regard, the SeV C proteins are unique in that they have been shown to occur in the cytoplasm as well as at the plasma membrane and in the nucleus [21–23]. In SeV-infected cells, most C proteins are detected in the cytoplasm by immunofluorescence microscopy [21,22]. However, in experiments with overexpression of the C proteins, the longer C protein has been detected predominantly at the plasma membrane, and the N-terminal 23 amino acid region of the C protein acts as a membrane-targeting and membrane-anchoring signal [19,23]. In addition, we recently reported that the smaller Y proteins (Y1 and Y2) were predominantly detected in the nucleus, although no potential nuclear localization signal (NLS) sequence was found within the protein [23].

In this paper, we found a cluster of basic amino acids at position 149-157 of the Y1 protein important for its active nuclear localization, and suggested a functional relevance of the nuclear-localizing activity to its ability to inhibit IFN-induced gene expression.

## Materials and Methods

### Cells and antibodies

293T cells (human renal epithelial cells expressing the SV40 large T antigen; RIKEN BRC Cell Bank, Tsukuba, Japan) were maintained in Dulbecco’s minimum essential medium (DMEM; Invitrogen) supplemented with 10% fetal bovine serum (FBS; Biological Industries, Kibbutz, Israel) and penicillin-streptomycin (Invitrogen) at 37 C as described previously [20]. A polyclonal antibody (pAb) against the SeV C protein was kindly provided by A. Kato (National Institute of Infectious Diseases, Japan). A pAb against the green fluorescent protein (GFP) (sc-8334; Santa Cruz biotechnology, Santa Cruz, CA) was used according to the manufacturer’s directions.

### Plasmid Construction

Plasmids encoding SeV C and Y1 mutants (C-d2Y, Y1-dY2, Y1-d192, -d188, and -d184) in pCAGGS.MCS and a plasmid encoding SeV Y2 in the pKS vector have been described previously [23,24]. The other Y1 mutants (Y1-d170, -d160, -d150, -KMK149A3, -TER152A3, -WLR155A3, -TLI158A3, -RGE161A3, -KTK164A3, -LKD167A3, -K149A, -M150A, -K151A, -W155A, -L156A, -R157A, -T158A, -L159A, -I160A, and -T152R,TLI158A3) were generated by introducing point-mutations using an AMAP site-directed mutagenesis kit (Amalgaam, Japan), and inserted back into the pCAGGS.MCS vector. The N-terminally EGFP-fused C and Y1 mutants [EGFP-C, -C(1–23), -Y1, and Y1-d150] were generated using a standard PCR technique, and inserted into pEGFP-C1 (Clontech). Each position was numbered from the first amino acid of the C protein with 204 residues. A reporter plasmid, pISRE-EGFP, was constructed by replacing the luciferase gene in pISRE-Luc (Agilent Technologies) with the EGFP gene by standard PCR. The full-length cDNA clone of N-terminally hemagglutinin (HA)-tagged human Ran GTPase was amplified from a total RNA sample of 293T cells by using an RT-PCR technique with specific primers and subcloned into the pCAGGS.MCS. The dominant-negative (DN) form of Ran containing T24N mutation was generated using an AMAP mutagenesis kit. All mutations were confirmed by DNA sequencing.

### Immunofluorescence microscopy

293T cells cultured in 6-well plates containing glass coverslips were transfected with the indicated plasmids using the FuGENE HD transfection reagent (Roche Diagnostics). At 24 h post-transfection (p.t.), cells were fixed with a 3% formaldehyde solution, and treated with 0.1% Triton X-100 in phosphate-buffered saline (PBS). Cells were then stained using anti-C pAb as a primary antibody and Alexa 488-conjugated anti-rabbit IgG goat pAb (Invitrogen) as a secondary antibody. The coverslips were mounted on glass slides and observed under a Zeiss LSM 5 confocal microscope (Carl Zeiss).

### Subcellular fractionation

293T cells cultured in 6-well plated were transfected with the indicated plasmids using the FuGENE HD reagent. At 24h p.t., subcellular fractions were prepared using a Nuclear/Cytosol Fractionation Kit (BioVision) according to the manufacturer’s directions.

### Reporter assay

293T cells cultured in 6-well plates were co-transfected with the indicated plasmids using the FuGENE HD reagent. At 18 h p.t., the culture medium was replaced with fresh medium containing IFN-α (1,000 IU/ml, R&D Systems). After an additional 8-h incubation, cells were lysed in SDS-PAGE sample buffer (125 mM Tris-HCl [pH 6.8], 4.6% SDS, 10% 2-mercaptoethanol, 0.005% bromophenol blue, and 20% glycerol) and analyzed by SDS-PAGE (12%) followed by Western blotting using pAbs against GFP and the SeV C protein as primary antibodies, and horseradish peroxidase (HRP)-conjugated anti-rabbit IgG goat pAb as a secondary antibody. Protein bands were visualized using an Immobilon Western Chemiluminescent HRP substrate (Millipore), and analyzed using a chemiluminescence imaging system (LAS-1000plus, Fuji Film).

## Results

### Smaller Y but not larger C proteins actively localize to the nucleus in a Ran GTPase-dependent manner

**Figure 1:**
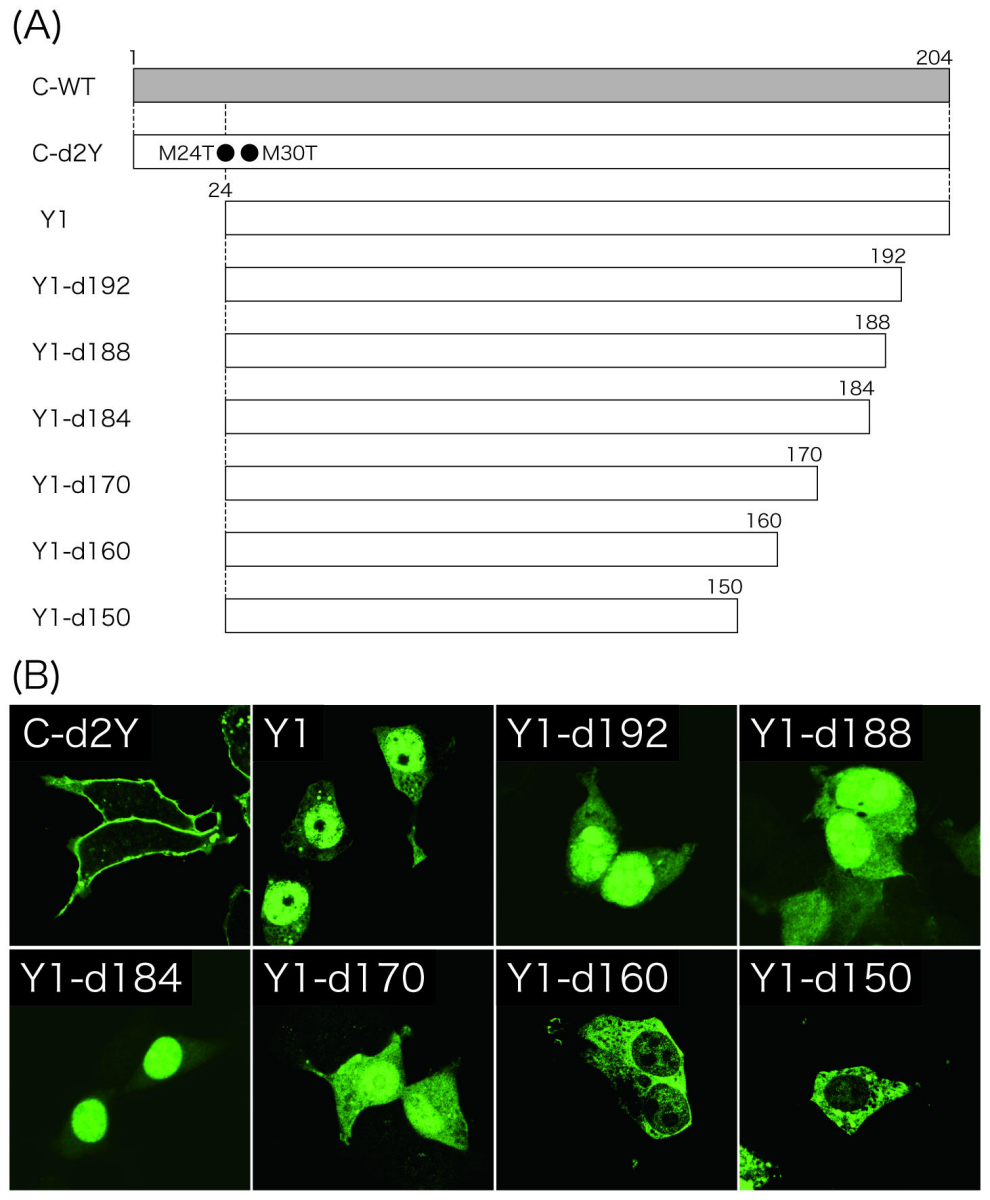
Subcellular localization of the Y1 mutants possessing C-terminal deletions. (A) Schematic representation of expression plasmids encoding C-WT, C-d2Y, Y1, Y1-d192, -d188, -d184, -d170, -d160, and -d150. (B) The C and Y1 proteins were expressed in 293T cells. At 24 h p.t., cells were fixed with 3% formaldehyde, permeabilized with 0.1% Triton X-100, and stained with anti-C pAb and Alexa 488-conjugated anti-rabbit IgG antibody as primary and secondary antibodies, respectively. Cells were observed under a Zeiss LSM5 confocal microscope.

We have previously reported that the overexpression of single proteins, SeV Y1 and Y2, lacking 23 and 29 amino acids from the N-terminus of the C protein, respectively, resulted in a subcellular distribution in the cytosol and/or nucleus, while the full-length C proteins (C-WT and C-d2Y, lacking expression of Y1 and Y2) were found predominantly at the plasma membrane (Figure 1B) [23]. A computer analysis could find no potential NLS throughout the C sequence (data not shown).

First, to identify the amino acid region responsible for the nuclear localization of the Y1 protein, the subcellular localization of a series of Y1 mutants possessing C-terminal deletions was analyzed by immunofluorescence microscopy using anti-C pAb (Figure 1). The Y1 mutants, Y1-d192, -d188, -d184, -d170, -d160, and -d150, were constructed by introducing stop codons at the positions next to the indicated numbers. In the Y1-d160 and -d150-transfected cells, fluorescence was no longer detected in the nucleus, while diffuse cytoplasmic and nuclear labeling was observed in the cells transfected with either Y1-d192, -d188, -d184, or -d170 (Figure 1B), indicating that the region from a.a. 150 to 170 of the Y1 protein is important to its nuclear localization. Among these Y1 mutants, Y1-d184 seemed to be localized in the nucleus in a greater degree than the other longer mutants including Y1. Since the C-terminal region of the C and Y proteins has been shown to be critical for their inhibitory effect against the Jak/STAT pathway, this might imply some functional difference between these proteins.

**Figure 2:**
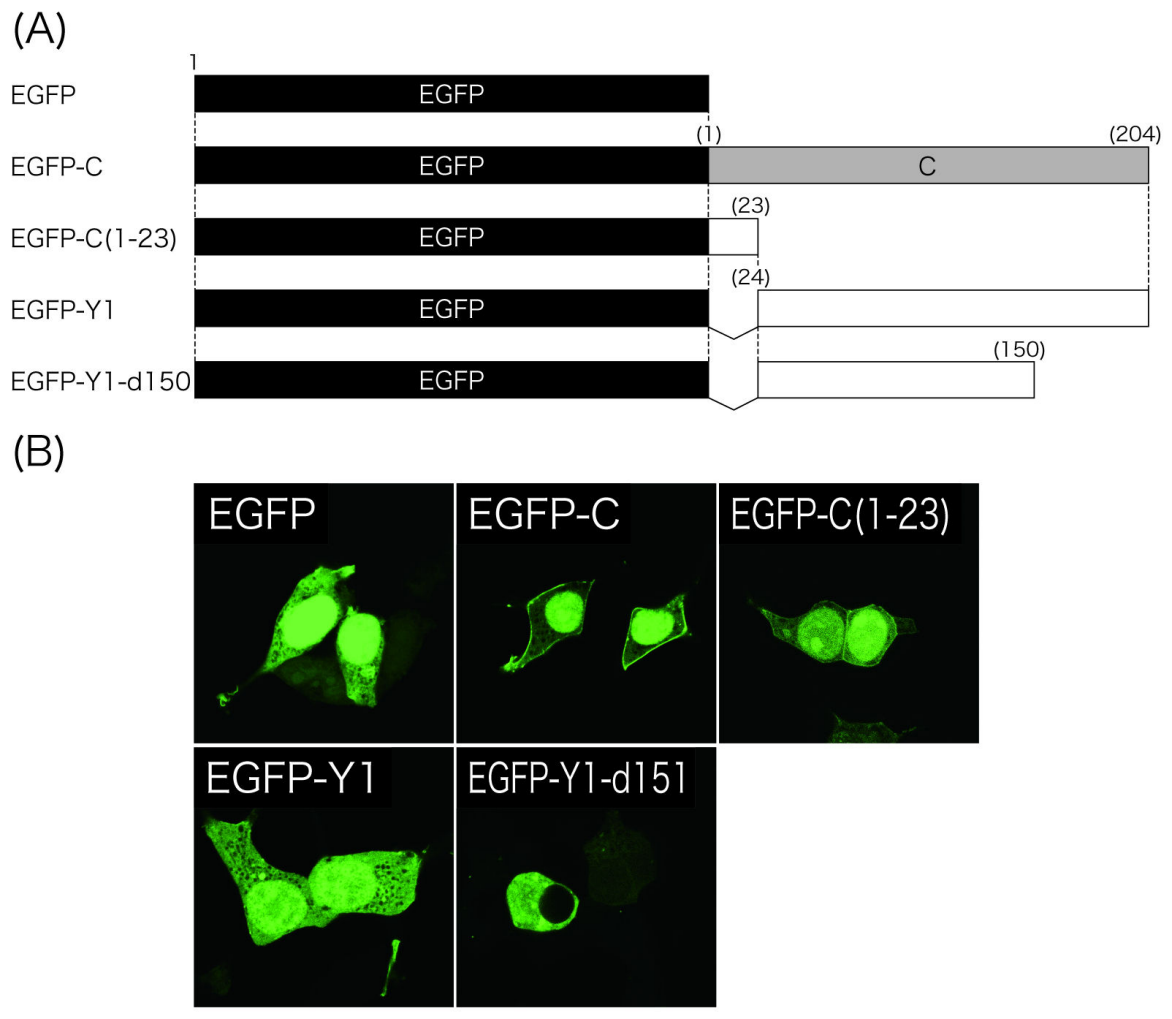
Subcellular distribution of the EGFP-fused C and Y1 mutants. (A) Schematic representation of expression plasmids encoding EGFP, EGFP-C, -C(1–23), -Y1, and -Y1-d150. (B) The EGFP-fused proteins were expressed in 293T cells. At 24 h p.t., cells were fixed with 3% formaldehyde and observed under a confocal microscope.

The nuclear pore complex allow unregulated passive diffusion of small molecules (< 20-40 kDa), but those of more than 40-60 kDa cannot migrate into the nucleus unless they contain NLSs [25]. Therefore, the nuclear localization of the approximately 20-kDa Y1 protein might be due to passive diffusion, even though the nuclear localizing ability of the Y1 mutants were not size-dependent (Figure 1). To examine this possibility, N-terminally EGFP-fused C and Y1 proteins were constructed (Figure 2A), and their subcellular localization was observed (Figure 2B). The expression of EGFP alone exhibited diffuse cytoplasmic as well as marked nuclear localization as a result of passive diffusion due to its small size of 27 kDa. In the cells transfected with the EGFP-C, in which the entire C protein was fused with the C-terminal of EGFP, marked nuclear fluorescence was still observed, but the fluorescence was detected also at the cell periphery, as expected from the ability of the C protein to preferentially localize at the plasma membrane. Interestingly, a similar distribution was observed in the cells transfected with the EGFP-C(1–23), in which the PM-targeting and membrane-anchoring N-terminal 23 amino acids of the C protein were fused to the C-terminal rather than N-terminal of EGFP. In the cells transfected with the N-terminally EGFP-fused Y1, EGFP-Y1, both diffuse cytoplasmic and nuclear localization was detected as in the Y1-transfected cells. In contrast, like Y1-d150, EGFP-Y1-d150, in which Y1-d150 was fused with the C-terminal of EGFP, could no longer be detected in the nucleus. Unlike the unmodified EGFP that localized both in the cytoplasm and nucleus, EGFP-Y1-d150 was found exclusively in the cytoplasm provably due to its increase in size. These results again confirm the nuclear localization of Y1 was not a result of passive diffusion.

**Figure 3:**
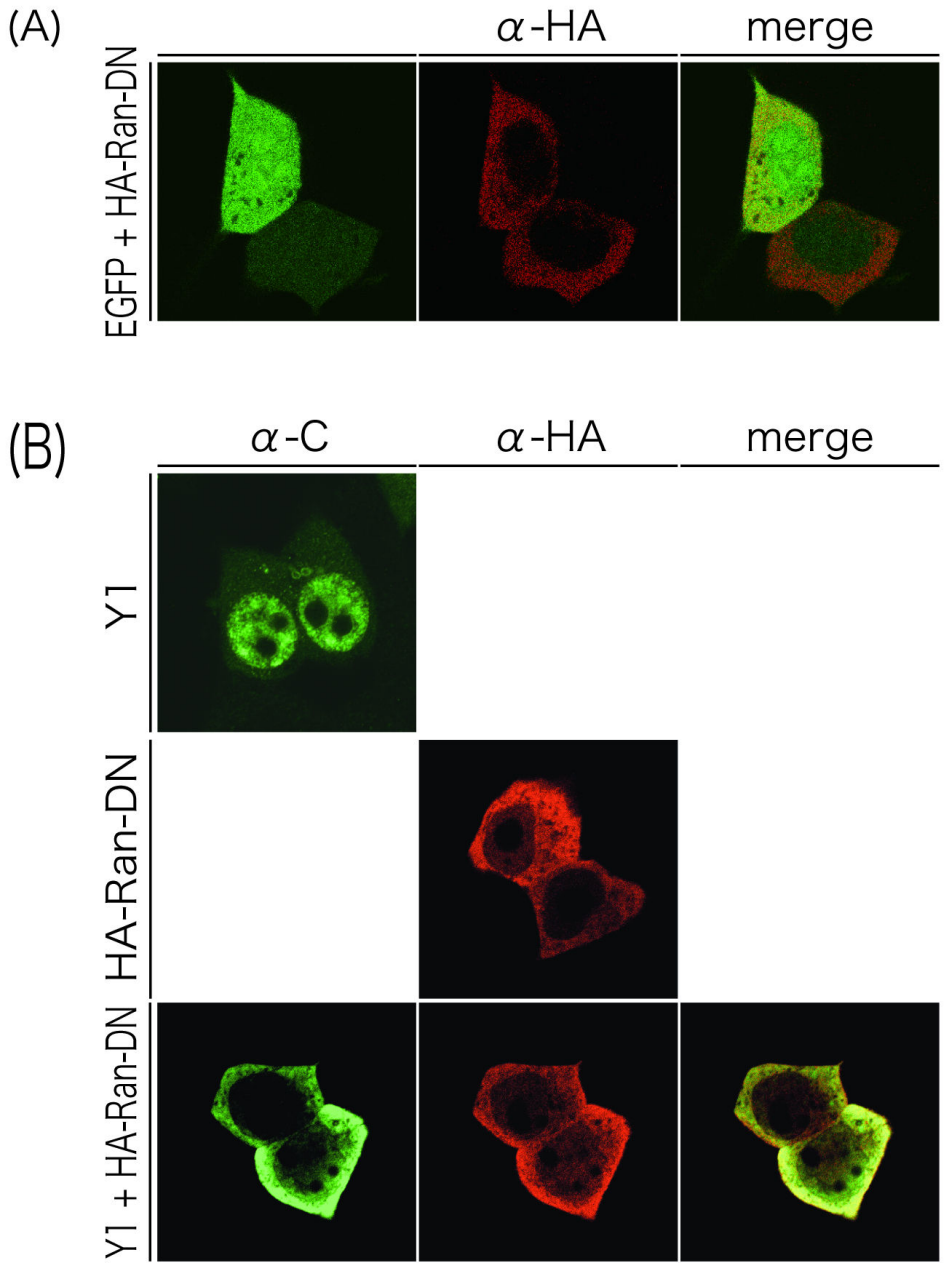
Subcellular distribution of EGFP (A) and the Y1 protein (B) in the presence of HA-Ran-DN. 293T cells co-transfected with Y1 and HA-Ran-DN were stained with anti-C (green) and anti-HA (red) antibodies at 24 h p.t., and observed under a Zeiss LSM5 confocal microscope.

**Figure 4:**
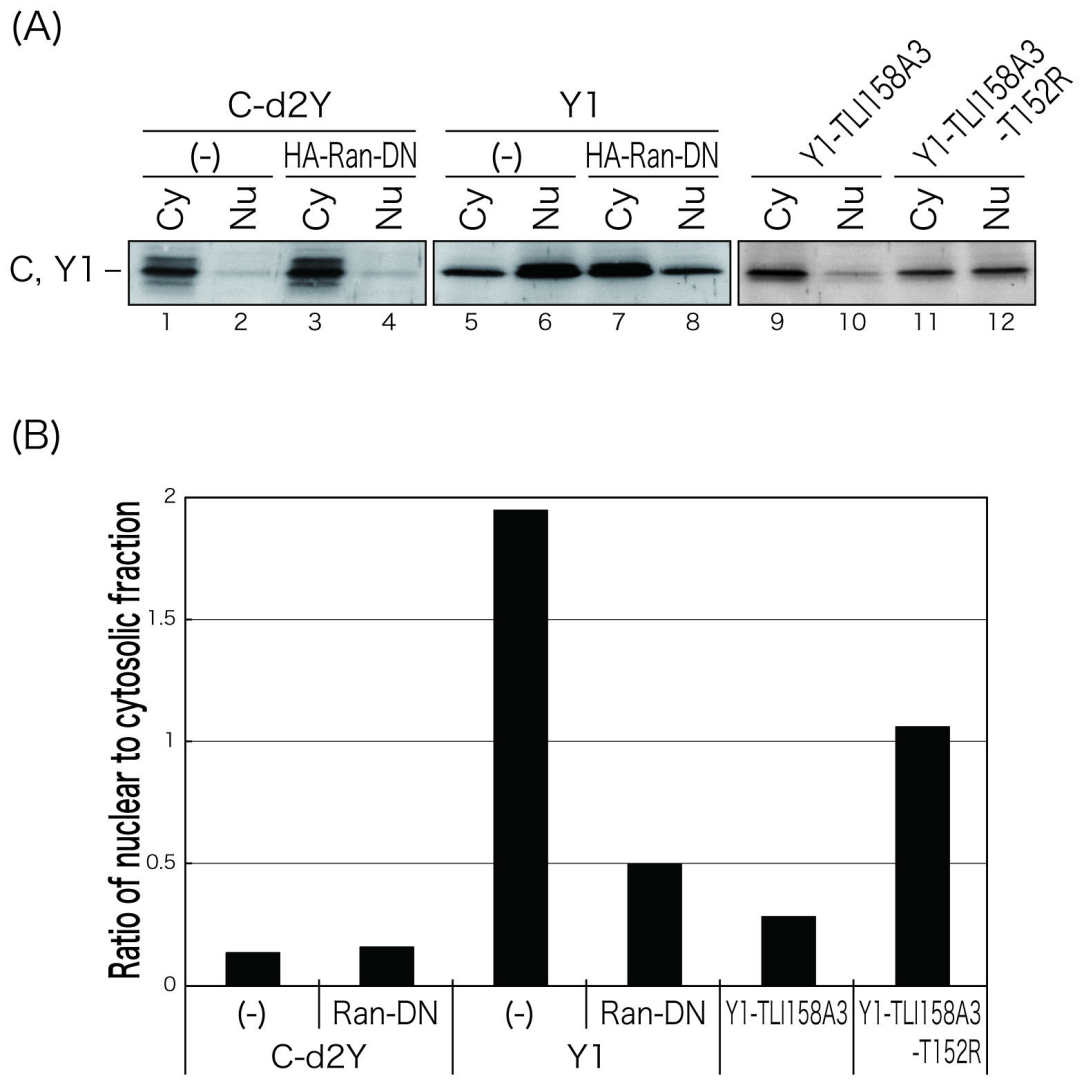
Fractionation analysis of 293T cells co-transfected with the C and Y1 mutants, and HA-Ran-DN. (A) Cytosolic (Cy) and nuclear (Nu) fractions were prepared as described in Materials and Methods at 24 h p.t., and equal amounts of each sample were analyzed by Western blotting using anti-C pAb. (B) The C and Y1 mutants in the cytosolic and nuclear fractions were quantitated using an LAS-1000 luminescent image analyzer. Ratios of the amounts each protein in the nuclear fractions to those in the cytosolic fractions are shown as bar graphs.

It has been known that Ras-related nuclear protein (Ran) is a GTPase involved in transport of proteins across the nuclear envelope, and a T24N mutation within Ran leads to a dominant-negative (DN) phenotype [26,27]. To further confirm that the Y1 protein actively transported to the nucleus but not by passive diffusion, we examined the effect of HA-tagged DN form of Ran (HA-Ran-DN) expression on the nuclear localization of Y1 (Figure 3). HA-Ran-DN alone expressed mostly in the cytoplasm (Figure 3B, middle panel). Co-expression of HA-Ran-DN with Y1 dramatically diminished the nuclear concentration of the Y1 protein (Figure 3, compare upper and lower panels), while nuclear distribution of EGFP was not affected by the expression of HA-Ran-DN (Figure 3A). This effect of HA-Ran-DN expression on the loss of nuclear localization of Y1 was further confirmed by fractionation of cells into cytosolic and nuclear extracts (Figure 4). C-d2Y observed at the plasma membrane and in the cytosol [19,23] was exclusively detected in the cytosolic fractions regardless of the presence or absence of HA-Ran-DN (Figure 4A, lanes 1-4 and 4B, left two bars). Consistent with Figure 3, the Y1 protein was detected in the cytosol more than in the nucleus in the presence of HA-Ran-DN, as opposed to the case in the absence of HA-Ran-DN where Y1 was detected in the nucleus more than in the cytosol (Figure 4A, lanes 5-8 and 4B, center two bars).

These results indicate that the Y1 protein has the ability to be actively distributed to the nucleus in a Ran GTPase-dependent manner, and a region of a.a. 150 -170 is responsible for that.

### Basic Amino Acids in the a.a. 149 -157 Region of the Y1 Protein Are Critical for Its Nuclear Localization

**Figure 5:**
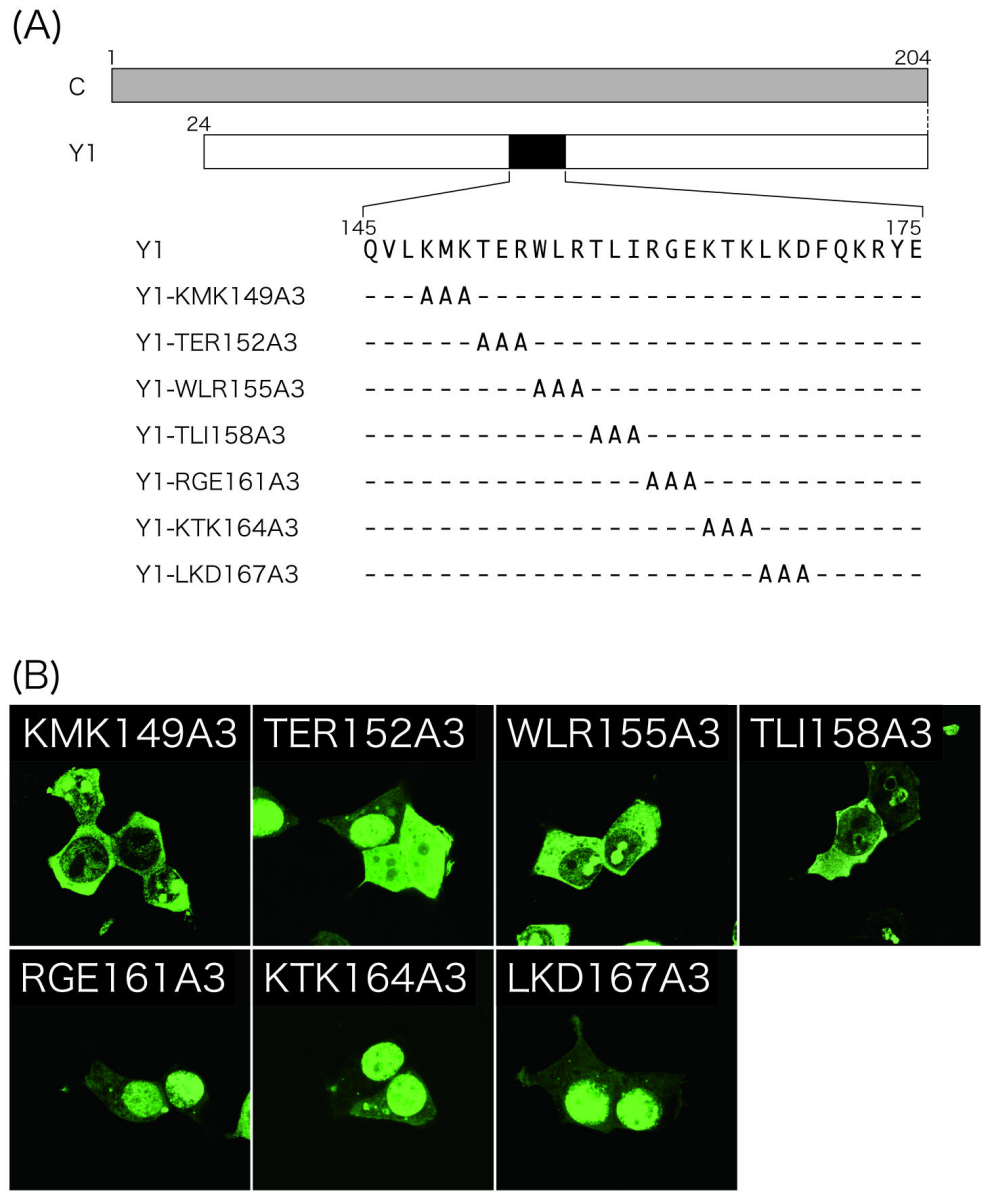
Subcellular distribution of the Y1 mutants possessing triple alanine substitutions. (A) Schematic representation of expression plasmids encoding Y1-KMK149A3, -TER152A3, -WLR155A3, -TLI158A3, -RGE161A3, -KTK164A3, and -LKD167A3. (B) The indicated mutants were subjected to immunofluorescent microscopy as shown in Figure 1B.

As mentioned above, no possible NLS was predicted throughout the C protein, despite its active transport to the nucleus. To identify amino acids critical for the nuclear localization of the Y1 protein, another series of Y1 mutants, Y1-KMK149A3, -TER152A3, -WKR155A3, -TLI158A3, -RGE161A3, -KTK164A3, and -LKD167A3, in which amino acid triplets starting from the indicated numbers within the 149-169 region were replaced by alanines, were constructed (Figure 5A). As for the Y1 protein, diffuse cytoplasmic and concentrated nuclear labeling was observed in the cells transfected with Y1-RGE161A3, -KTK164A3, and -LKD167A3. In the Y1-TER152A3-expressing cells, the cytoplasmic fluorescence was more apparent, but nuclear fluorescence was still readily detectable. In contrast, cytoplasmic labeling was clearly detected, but nuclear fluorescence was no longer detected in the cells expressing Y1-KMK149A3, -WLR155A3, and -TLI158A3 (Figure 5B).

**Figure 6:**
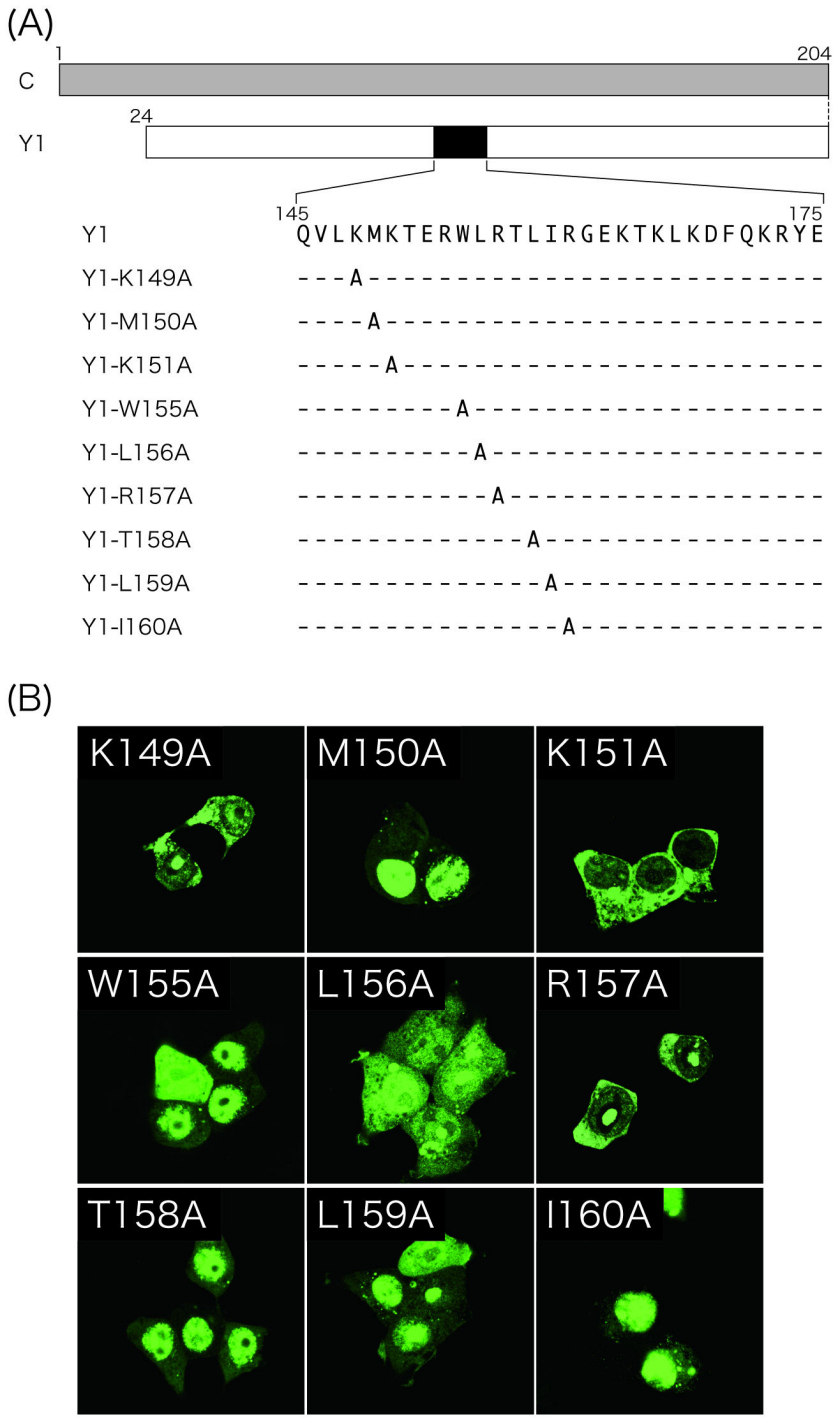
Subcellular distribution of the Y1 mutants possessing single alanine substitutions. (A) Schematic representation of expression plasmids encoding Y1-K149A, -M150A, -K151A, -W155A, -L156A, -R157A, -T158A, -L159A, and -I160A. (B) The indicated mutants were subjected to immunofluorescent microscopy as shown in Figure 1B.

To make a short list of the amino acids responsible for the nuclear localization of the Y1 protein, single substitutions with an alanine were introduced into the KMK149, WLR155, and TLI158 regions (Figure 6A). The substitutions of K149A, K151A, and R157A resulted in a loss of the nuclear fluorescence, while the others did not greatly affect the subcellular localization of Y1 (Figure 6B). These results suggest that the basic K149, K151, and R157 residues are important for nuclear localization of the Y1 protein, just as basic amino acids play a key role in NLS function.

**Figure 7:**
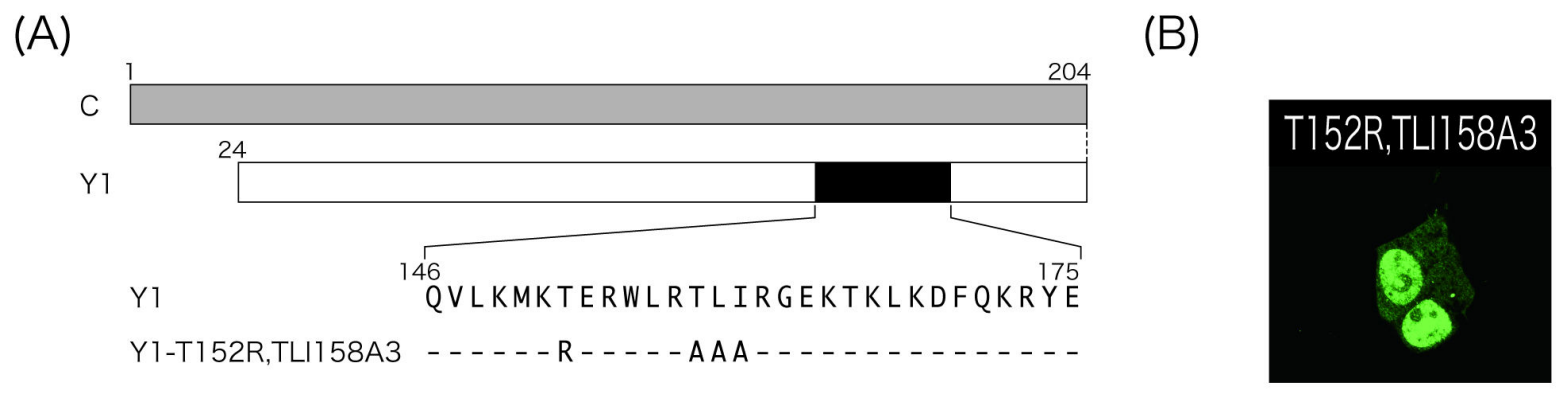
Subcellular distribution of the Y1 mutant, Y1-T152R,TLI158A3. (A) Schematic representation of Y1-T152R,TLI158A3. (B) Cells expressing the mutants were subjected to immunofluorescent microscopy as shown in Figure 1B.

To firmly verify the importance of basic amino acids to the nuclear-localizing function of the Y1 protein, we examined whether the loss of nuclear localization of Y1-TLI158A3 could be recovered by the substitution of a non-basic threonine with a basic arginine residue at position of 152 (Figure 7A). In the cells transfected with the mutant, Y1-T152R,TLI158A3, predominant nuclear fluorescence was observed, although the parental Y1-TLI158A3 had lost the ability for nuclear localization (compare Figures 5B and 7B). This alteration of the ability of nuclear localization was further confirmed by a fractionation analysis (Figure 4A, lanes 9-12 and 4B, right two bars).

These results indicate that basic amino acid residues within the 149-157 region of the Y1 protein play an important role in its nuclear localizing ability, although an adjacent, non-basic amino acid triplet TLI158 is able to affect the function.

### Nuclear localization of the Y1 protein parallels its antagonizing-ability against IFN-α-induced gene expression

**Figure 8:**
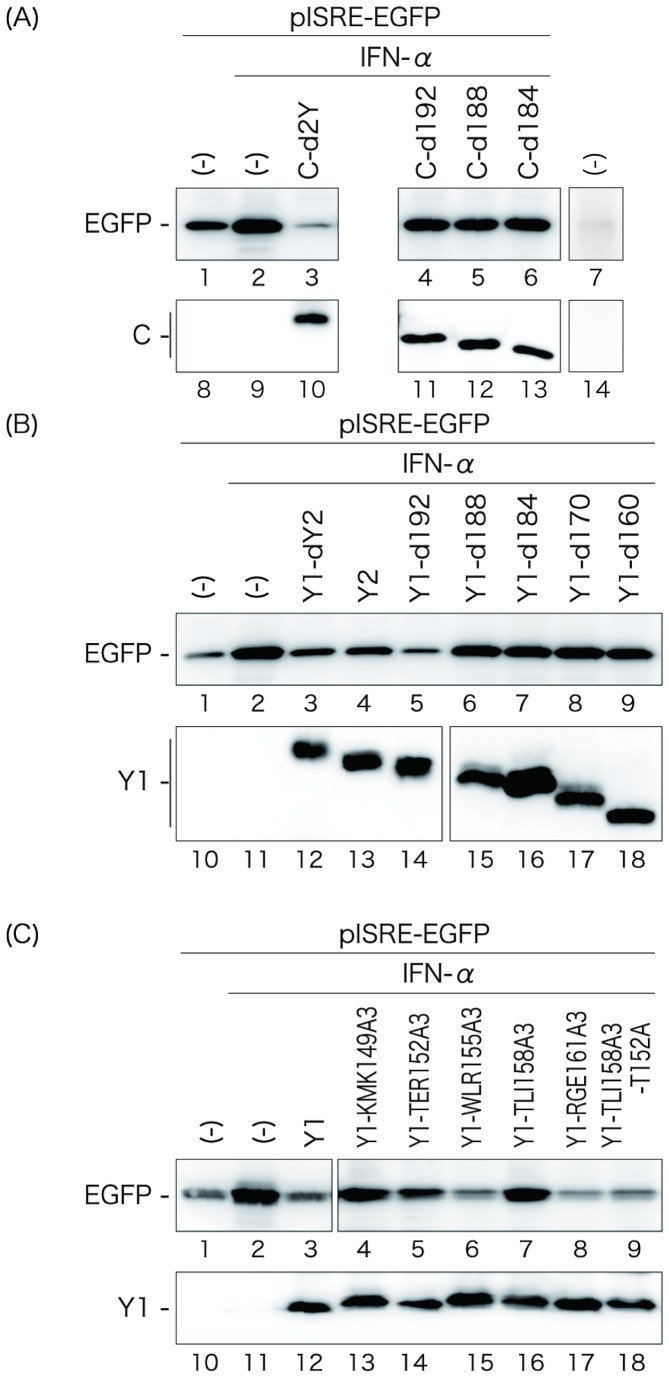
ISRE reporter assay in the presence of the C and Y mutants. 293T cells were co-transfected with a series of deletion mutants of the C protein (A), deletion mutants of the Y1 protein (B), or the Y1 mutants possessing triple alanine substitutions (C) together with a reporter plasmid, pISRE-EGFP. At 18 h p.i., cells were treated with IFN-α (1,000 IU/ml) for 8 h, and then expression level of EGFP and the transfected C and Y1 proteins were analyzed by Western blotting using anti-GFP and anti-C antibodies.

The best-known function of the SeV C proteins is the counteraction of IFN-induced antiviral responses. To find a relevance of the nuclear localization of the Y1 protein to its antagonism of IFN-signaling, we finally examined whether the Y1 mutants would be able to disrupt IFN-induced reporter gene expression. 293T cells were co-transfected with the indicated C or Y1 mutants together with a reporter plasmid, pISRE-EGFP, in which expression of EGFP was controlled by an IFN-stimulated response element (ISRE), and then treated with IFN-α to induce EGFP expression, as performed previously [28–30]. The expression level of EGFP in each sample was compared and expression of the C and Y mutants was confirmed by Western blotting using anti-GFP and anti-C pAbs, respectively (Figure 8). In the absence of any of the C and Y proteins, expression of EGFP induced by IFN-α treatment was readily detectable by Western blotting using anti-GFP pAb (Figure 8A-C, lanes 2).

As for the C protein, expression of the 204-residue-long C protein clearly inhibited EGFP expression (Figure 8A, lane 3), but the C-terminal deletions of 12-20 amino acids of the C protein caused significant loss of the inhibition (Figure 8A, lanes 4-6).

As expected from previous reports [13], the Y1-dY2 lacking expression of Y2 and Y2 proteins were also able to reduced EGFP expression (Figure 8B, lanes 3 and 4). Like the C deletion mutants, the Y1 mutants possessing C-terminal deletions of more than 16 amino acids no longer inhibited EGFP expression regardless of whether they were nuclear-localizing or not (Figure 8B, lanes 6-9). Interestingly, unlike C-d192, Y1-d192 possessing a deletion of C-terminal 12 amino acids was still able to inhibit EGFP expression (Figure 8B, lane 5), implying that the mechanism for inhibition of IFN-induced gene expression might be different between the C and Y1 proteins.

More interestingly, as for the Y1 mutants possessing triple amino acid substitutions, their ability to inhibit EGFP expression almost correlated with that to localize in the nucleus (Figure 8C). Indeed, the nuclear-localizing Y1-RGE161A3 was able to inhibit EGFP expression at a level comparable to the Y1 protein (Figure 8C, lane 8), but all of the non-nuclear-localizing mutants failed to inhibit the expression with the exception of Y1-WLR155A3 (Figure 8C, lanes 4, 6, and 7). Consistent with the increased cytoplasmic fluorescence, the level of inhibitory effect of Y1-TER152A3 on IFN-induced EGFP expression was reduced compared to that observed in the cells expressing the other nuclear-localizing Y1 proteins (Figure 8C, lane 5). In addition, consistent with the recovered nuclear localization observed in the Y1-T152R,TLI158A3-expressing cells, loss of the ability of Y1-TLI158A3 to inhibit reporter EGFP expression was also recovered by an additional T152R mutation (Figure 8C, lanes 8 and 9).

These results suggest that the ability of the Y1 protein to localize in the nucleus may be relevant to that to inhibit IFN-induced gene expression.

## Discussion

P mRNA of most of the paramyxoviruses encode multiple polypeptides: P as well as two major accessory proteins, C and V, were translated from the intact and/or edited P mRNAs [1]. The best-characterized function of these accessory proteins is to counteract host innate immunity. In this regard, SeV has the most diverse functions [2]. The V protein has been shown to inhibit retinoic acid-inducible gene I (RIG-I)-like receptor (RLR)-dependent signaling leading to the activation of interferon regulatory factor-3 (IRF3) followed by IFN-β production through physical interaction not only with one of the RLRs, melanoma differentiation-associated gene 5 (MDA5), but also with interferon regulatory factor-3 (IRF-3) [28,31–33].

The C protein has also been reported to prevent the induction of the host innate immune responses by interfering with multiple steps of the pathways. Its best-known function is the counteraction of the well-characterized IFN-α/β signaling (Jak/STAT) pathway via inhibition of tyrosine phosphorylation of STAT2 through interaction with STAT1 or by inducing instability of STAT1 in certain types of cells, resulting in suppression of ISG activation and subsequent establishment of an antiviral state among the host cells [11,14,15,17]. In addition, the C protein has been known to block also the IFN-γ signaling pathway by preventing interaction of gamma-activated factor (GAF), a homodimer of phosphorylated STAT1, and gamma-activated sequence (GAS) in the promoter of ISGs in the nucleus [3,5,13,16].

The C protein seems to play roles not only in inhibition of the IFN-signaling pathways but also in prevention of the RLR-dependent IFN-inducing pathway. The C protein is reported to have the ability to limit the generation of viral double-stranded (ds) RNA [20,34]. This limitation is suggested to keep protein kinase R (PKR) inactive, which is activated by the binding of dsRNA and induce antiviral actions of the host cells [34]. Moreover, the C protein inhibits RLR-dependent activation of IRF3 and induction of IFN-β by an unknown mechanism [20,35].

In addition to counteracting host innate immunity, the C protein plays multiple roles in the course of viral replication, and has been shown to inhibit apoptosis induced by viral infections, promote viral assembly and budding through interaction with Alix/AIP1, and regulate polarized viral RNA synthesis for efficient production of infectious viral particles [4,6,20,23,24,36–40]. Perhaps reflecting such diverse functions, the C protein is able to localize at various sites in the cell, which might be the appropriate place to exert its functions. In the SeV-infected cultured cells, the C protein was mainly detected in the cytoplasm by immunofluorescence microscopy [21,22]. However, in the case of a single protein expression from cDNA, the C protein localized predominantly at the plasma membrane, since the N-terminal 23 amino acid region has been shown to serve as membrane-targeting and membrane-anchoring signals [19,23]. The localization of the C protein at the plasma membrane has been reported to be important for the IFN-independent phosphorylation of STAT1, although the significance of this phosphorylation is unknown, and for promoting budding of viral and virus-like particles by recruiting cellular ESCRT machinery along with Alix to the site of budding on the plasma membrane [19,23].

In this paper, we showed that the smaller Y1 protein, lacking the N-terminal membrane-targeting and membrane-anchoring signal region, was able to actively localize to the nucleus in a Ran GTPase-dependent manner. The clustered basic amino acids within the 149-157 region were critical for the nuclear localizing function, consistent with the notion that the basic residues are critical for the function of known NLSs, although no potential NLS was found throughout the protein. Nuclear localization of the Y1 protein seems to be relevant to its ability to inhibit IFN-stimulated activation of ISGs, since these two abilities almost paralleled each other with an exception for Y1-WLR155A3, which retained the ability to inhibit the Jak/STAT signaling pathway despite the loss of nuclear localization. However, it might be impossible to connect a single region with a single function of the C proteins, since it has been reported that multiple amino acids widely spread in the C protein are involved in its inhibitory effect against the IFN-signaling pathway, although the C-terminal half is enough and the C-terminal end of the C protein is essential for the function [12,13,17,20].

Interestingly, the mechanism for inhibition of the IFN-signaling pathway seems to be different between the C and Y1 proteins, since the C-terminal deletion of 12 amino acids resulted in the loss of IFN-antagonizing ability for the C, but not Y1, protein (Figure 8). It has been reported that the C-terminal half (a.a. 85 -204) of the C protein is enough for the interaction with STAT1 as well as inhibition of IFN-mediated induction of ISGs and subsequent establishment of the antiviral state, and thus, both the C and Y1 proteins retain all of these abilities [12,13]. Blockade of the INF-α/β-signaling pathway might be achieved by both interaction of the C protein with STAT1 in the cytoplasm and that of the Y1 protein in the nucleus.

Conflicting with the above observations, there are other reports using MEFs and 2fTGH cells, that the Y1 protein possesses the ability to inhibit IFN-signaling, but not the establishment of an antiviral state, while only the full-length C protein is able to inhibit both [11]. This conflict might imply the presence of a parallel pathway independent of the Jak/STAT pathway, and the different subcellular localization of the C and Y1 proteins might reflect differences in their targeting pathways.

As described above, the C protein is able to block not only the IFN-α/β but also the IFN-γ signaling pathways [3,5,13,16]. Although the detailed mechanism has not been revealed yet, it has been reported that the C protein is not able to prevent IFN-γ-mediated tyrosine and serine phosphorylation of STAT1 and subsequent translocation of the phosphorylated STAT1 into the nucleus, but is able to prevent GAF-GAS interaction in the nucleus [16,41]. Since the Y1 protein is able to bind to STAT1, the blockade of the IFN-γ pathway might result from the Y1-GAF interaction in the nucleus.

Experiments to elucidate the detailed mechanism for blockade of the IFN-signaling pathways by the C and Y1 proteins are currently underway using *in vitro* cDNA-based expression systems as well as SeV recombinants possessing amino acid substitutions within the C protein which cause a defect of the nuclear-localizing ability. Further understanding of viral strategies for escape from host innate immunity will hopefully lead to a better understanding of viral pathogenesis and the development of novel therapeutics to target important steps in viral replication.
